# Healthcare attention and access for deaf individuals: a phenomenological study

**DOI:** 10.15649/cuidarte.4665

**Published:** 2025-09-12

**Authors:** Daniel Minte-Valderrama, Valentina Silva-Zurita, Camila Alvarado-Salfate, Jenifer Villa-Velásquez, Miguel Valencia-Contrera, Flérida Rivera-Rojas

**Affiliations:** 1 Universidad Austral de Chile, School of Nursing. Puerto Montt, Chile. E-mail: d.minte@live.cl Universidad Austral de Chile Puerto Montt Chile d.minte@live.cl; 2 Universidad Austral de Chile, School of Nursing. Puerto Montt, Chile. E-mail: valesilvazu@gmail.com Universidad Austral de Chile Puerto Montt Chile valesilvazu@gmail.com; 3 Universidad Austral de Chile, School of Nursing. Puerto Montt, Chile. E-mail: cami.isabel27@gmail.com Universidad Austral de Chile Puerto Montt Chile cami.isabel27@gmail.com; 4 Universidad Austral de Chile, Escuela de Enfermería. Puerto Montt, Chile. E-mail: jenifer.villa@uach.cl Universidad Austral de Chile Puerto Montt Chile jenifer.villa@uach.cl; 5 Universidad Andrés Bello, Nursing Faculty. Santiago, Chile. E-mail: miguel.valencia@unab.cl Universidad Andrés Bello Santiago Chile miguel.valencia@unab.cl; 6 Universidad Católica del Maule, Nursing Department. Curicó, Chile. E-mail: frivera@ucm.cl Universidad Católica del Maule Curicó Chile frivera@ucm.cl

**Keywords:** Persons with Hearing Impairments, Vulnerable Populations, Health Services Accessibility, Effective Access to Health Services, Barriers to Access of Health Services, Personas con Deficiencia Auditiva, Poblaciones Vulnerables, Accesibilidad a los Servicios de Salud, Acceso Efectivo a los Servicios de Salud, Barreras de Acceso a los Servicios de Salud, Pessoas com Deficiência Auditiva, Populações Vulneráveis, Acessibilidade aos Serviços de Saúde, Acesso Efetivo aos Serviços de Saúde, Barreiras ao Acesso aos Cuidados de Saúde

## Abstract

**Introduction:**

In Chile, access to health care for deaf individuals faces communication and legal barriers.

**Objective:**

To reveal the experiences of deaf people from a southern province of Chile regarding their health care access between February 2022 and February 2023.

**Materials and Methods:**

A qualitative phenomenological study was conducted with deaf individuals who had accessed health services in the past year. Semi-structured personal interviews were conducted with the support of Chilean Sign Language interpreters.

**Results:**

Participants aged 24 to 30 reported communication difficulties with health personnel, exacerbated by the lack of interpreters or facilitators trained in sign language, forcing them to rely on third parties and technological aids.

**Discussion:**

The barriers identified in health care access align with existing literature; however, the limited number of studies on the Chilean context restricts local comparisons.

**Conclusion:**

The findings highlight access barriers and issues in health care delivery, impacting care quality. Key challenges include improving physical spaces, communication strategies, health literacy, training, and culturally competent care.

## Resumen

**Introducción:**

En Chile, el acceso a la salud de personas sordas enfrenta barreras comunicacionales y legales.

**Objetivo:**

Develar las vivencias de personas sordas pertenecientes a una provincia del sur de Chile, respecto a la atención y acceso a los servicios de salud entre los meses de febrero 2022 y febrero del 2023.

**Materiales y métodos:**

Estudio cualitativo fenomenológico, en personas sordas que acudieron a un servicio de salud en el último año. Se realizaron entrevistas personales semiestructuradas, con el acompañamiento de intérpretes en lengua de señas chilena.

**Resultados:**

Los participantes tenían entre 24 y 30 años, quienes mencionaron dificultades comunicativas con el personal de salud, acrecentadas por la falta de intérpretes o facilitadores capacitados en lengua de señas, siendo forzados a solicitar ayuda de terceros y apoyos tecnológicos.

**Discusión:**

Las barreras identificadas en la atención y el acceso a la salud de las personas son coincidentes con el estado del arte, sin embargo, debido al escaso número de estudios realizados en la realidad chilena, es limitada la comparación local de los hallazgos.

**Conclusiones:**

Las vivencias relevan barreras en el acceso y problemas en la atención en salud, lo que repercute en la calidad del cuidado, destacando desafíos relacionados a la mejora de espacios físicos y de encuentro, estrategias de comunicación y atención, alfabetización, capacitación y cuidados culturalmente competentes.

## Resumo

**Introdução:**

No Chile, o acesso à saúde para surdos enfrenta barreiras legais e de comunicação.

**Objetivo:**

Revelar as experiências de surdos em uma província do sul do Chile em relação aos cuidados de saúde e ao acesso aos serviços entre fevereiro de 2022 e fevereiro de 2023.

**Materiais e métodos:**

Um estudo qualitativo fenomenológico foi conduzido com surdos que frequentaram um serviço de saúde no último ano. Entrevistas pessoais semiestruturadas foram realizadas com o auxílio de intérpretes de Língua de Sinais Chilena.

**Resultados:**

Os participantes tinham entre 24 e 30 anos e relataram dificuldades de comunicação com os profissionais de saúde, agravadas pela falta de intérpretes ou facilitadores treinados em língua de sinais, forçando-os a buscar ajuda de terceiros e suporte tecnológico.

**Discussão:**

As barreiras identificadas aos cuidados de saúde e ao acesso para os indivíduos são consistentes com o estado da arte; no entanto, devido ao número limitado de estudos realizados no Chile, as comparações locais dos resultados são limitadas.

**Conclusões:**

As experiências revelam barreiras de acesso e problemas na assistência à saúde, que impactam a qualidade do atendimento, destacando desafios relacionados à melhoria dos espaços físicos e de reunião, estratégias de comunicação e cuidado, alfabetização, treinamento e atendimento culturalmente competente.

## Introduction

According to the World Health Organization (WHO), more than 5% of the world’s population suffers from a disabling hearing loss, of which nearly 80% live in low- and middle-income countries^1^. In Chile, according to the Third National Disability Study^2^, the percentage of adults with deafness is 4%, while the percentage of children and adolescents with deafness or hearing difficulties is 1.10%. 

Within the Chilean legal framework, Law 20.422 defines a deaf person as“someone who, due to reduced or non-existent auditory functionality, acquired either from birth or throughout their life, has developed as a predominantly visual individual, with the right to access and use sign language, to possess a deaf culture, and to identify as a member of a minority linguistic and cultural community”^3^. In this regard, Chilean Sign Language (LSCh, by its initials in Spanish) is considered the natural and original language, as well as an intangible heritage of deaf individuals in Chile, and an essential element of their culture and identity. 

On the other hand, this legal framework establishes the State’s obligation to promote, respect, and safeguard the cultural and linguistic rights of deaf individuals, ensuring their access to public and private services, education, the labor market, health care, and other areas of social life in sign language^3^. In line with this, according to the United Nations^4^, the right to health involves, among other elements, “accessibility,” which stipulates that services, goods, and health facilities must be accessible to everyone without discrimination. Accessibility dimensions include non-discrimination, physical accessibility, economic accessibility, and access to information. 

In this context, in 2022, the Chilean Ministry of Health (MINSAL, by its initials in Spanish) published the “National Hearing Health and Ear Care Plan for Chile 2021–2030,”^5^ which sets health goals aimed at reducing existing gaps in health care. Nevertheless, Campos and Cartes-Velásquez point out that access to health care for deaf individuals in Chile has been mainly limited by communication barriers^6^, since “there is no legislation that protects access to health care in sign language.” 

Despite the great relevance of the phenomenon, few studies provide information on the Chilean reality from the perspective of the main stakeholders, as highlighted in a recent literature review in the field^7^. In this context, the present study emerges to address this issue, aiming to uncover the experiences of deaf individuals from a province in southern Chile regarding health care and access to health services. Furthermore, based on the aforementioned considerations, the assumptions guiding this study are that there are linguistic gaps in health care in Chile and that the existence of structural inequities within the Chilean health system limits care and access to health services for deaf individuals. 

## Materials and Methods

Considering the nature of the phenomenon of interest, a phenomenological design was chosen, drawing on Alfred Schütz’s perspective^8^, which emphasizes the subject in interaction within meaningful social structures. For the development of the study, the conceptual framework of the United Nations was adopted^4^, taking into account the dimensions of access to health care: non-discrimination, physical accessibility, economic accessibility, and access to information. 

The unit of analysis consisted of deaf individuals residing in a province in southern Chile. Regarding sample size, given the exploratory nature of the study and the complex access to the population, it was composed of five participants. 

The inclusion criteria were: residing in a province in southern Chile, having attended a health care service within the past year, and using LSCh or a personal sign language as the primary mode of communication. Exclusion criteria included: being under 18 years of age; being a person with hearing impairment (since this category encompasses conditions ranging from mild to profound hearing loss, with the potential use of cochlear implants or hearing aids); being a deaf individual who primarily uses oral language as their means of communication; or having another physical, mental, intellectual, or visual disability that would create an additional difficulty. 

Participants were contacted through a public call disseminated via a video posted on social media, which included immersive reading, audio, and sign language interpretation, the latter presented in an optimal size for visibility (over 25.00% of the screen). After establishing contact with potential participants and confirming the inclusion and exclusion criteria, the study procedures were explained to them, and they were provided with the informed consent form in both physical and digital formats, including immersive reading and LSCh interpretation.

Semi-structured interviews with open-ended questions were conducted regarding the factors surrounding the experience of attending a health care center. These were carried out with the support of a sign language interpreter, a professional proficient in LSCh, as well as spoken and written language, who interpreted messages from one language to another. The structure and content of the interview were reviewed by experts in both subject matter and methodology. 

Data collection was carried out through audio and video recordings of the interviews, along with field notes taken by the researchers. Data analysis was conducted concurrently with the collection process. Each interview was transcribed verbatim, and the information was segmented into units of analysis, which were classified according to themes or recurring patterns to be later categorized. These units of analysis were subjected to a coding process supported by the software ATLAS.ti, which facilitated the grouping of analysis units according to codes. Data analysis triangulation was performed in two phases by the research team, achieving consistency in the findings. All collected data are freely available for access and consultation in Mendeley Data^9^. 

The study was approved by a Scientific Ethics Committee (ORD. N°012, Letter of Favorable Report on Research Protocol) to ensure the validity of the study and its conduct in accordance with the ethical considerations of scientific research. 

## Results

The participants presented heterogeneous characteristics, with varying age ranges, educational levels, and socioeconomic backgrounds ( Table 1).

**Table 1:** Characteristics of the Participants

Informant Code	Gender	Age (years)	Occupation
E1	Male	24	Student
E2	Male	36	Teacher
E3	Male	32	Warehouse Worker
E4	Female	31	Homemaker
E5	Male	31	Teacher

From the data analysis, 55 relevant codes were identified, leading to the development of 12 categories. These categories were grouped by seeking points of convergence and new content similarities that would address the stated objectives, taking into account the theoretical elements of access (non- discrimination, physical accessibility, economic accessibility, and access to information) (see Table 2).

**Table 2:** Composition of Access Dimensions

Accessibility Dimension	Min
Access to Information	- Complementary means of communication - Communication problems between deaf individuals and health care personnel - Communication assistance needs of deaf individuals
Non-Discrimination	- Health professionals’ behavior toward deaf individuals - Emotional experiences of deaf individuals in relation to health care - Responses of deaf individuals to difficulties in health care - Perceived barriers in health care - Current state of the health care system - Inclusion of deaf culture - Compliance with current legislation
Proposals for Improvement	- Support for deaf individuals in health care - Adjustments suggested by deaf individuals

**Dimension: Access to Information**

Deaf individuals who use the Chilean health care system report difficulties throughout the entire care process, having to employ different methods to communicate. For example, some have been asked to use writing, which does not facilitate communication at all, since the grammar used in Chilean Sign Language differs significantly from that used in written Spanish.

*“Then I start to feel uncomfortable, I begin to write, they look at me, understand a little, explain to me, but there are words that I cannot understand; therefore, communication becomes very, very, very difficult” (E1, P12).*

Most of them must be accompanied or assisted via video call by third parties—whether family members, acquaintances, or coworkers—who must act as interpreters between the patient and the health care staff.

*“It would have been great, of course, to have someone present to facilitate communication, but through the phone […] considering how I felt with all the injections (peripheral infusion catheter) I had at that moment, right? I had one hand holding the phone and the other with the IV lines in my arm, so that issue was extremely difficult” (E2, P13).*

The sign language proficiency of companions can become an obstacle in the care of deaf individuals, as knowledge of everyday vocabulary is often insufficient in a health care context. Additionally, concerns arise regarding interpreter confidentiality due to the presence of value judgments, questioning, and a lack of privacy.

*“For example, it could be a case of a suicide attempt. What happens? I go with a friend or someone, and they might start criticizing me as a person instead of me receiving the service I need at that moment, which is for care. But obviously, that creates a problem” (E2, P27).*

Another difficulty related to the individual providing sign language interpretation is the availability of service hours, as interpretation is not offered 24 hours a day.

*“It was 1:00 a.m. How was I supposed to call if the interpreters were sleeping at 1 or 2 in the morning? How could I bother them? Obviously, that would have been an interruption, because we also have to respect their schedules. Of course, in the hospital they work day and night, but the people who provide interpretation services do not” (E2, P15).*

In their interactions with health care staff directly involved in their care, the interviewees highlighted communication problems that may pose risks to the patient’s health—whether in the process of obtaining a medical diagnosis, administering treatment, performing a procedure, or in relation to the patient’s need and right to know and understand information about their health condition.

*“So, there was no communication. I wrote to him, I told him which one it was, but the doctor didn’t understand and thought it was another one. So my tooth kept hurting, and then they realized they had extracted the wrong tooth” (E1, P15).*

*“Honestly, the relationship, the communication fails, because they just examine me and leave, so there is no information. […] When they want to inject me with something, I don’t know what it is, what the name of that vaccine is. I have to make them wait because I need them to first give me information about the procedure they are going to perform” (E5; P23, P25).*

**Dimension: Non-Discrimination**

The constant environmental barriers have led users to a variety of experiences, most of which are negative, such as anger, sadness, and a sense of detriment and impunity, as they feel that their complaints and requests for deaf culture to be considered are not heard.

*“It is their duty to ensure inclusion here, I feel that. The system must respect this in the hospital. And that’s when I start to feel a bit of anger, but I control myself and once again make an appointment, so I go to the doctor” (E5, P24).*

The interviewees made explicit the inequalities they perceived in health care, emphasizing feelings of exclusion and loneliness:

*“We are deaf people, we are alone, so how?” (E1, P11).*

They also described their perceptions regarding the behavior of some health care staff, highlighting a lack of empathy, ethics, and respect:

*“There is also disdain. They tell us we are equal, but it’s only words, because later, in their private thoughts, they believe they are superior, and there is disdain. It is a lack of respect” (E5, P33).*

The interviews revealed a sense of uncertainty due to the participants’ dependence on others to navigate the health care context, as well as a perception of an unfavorable future regarding progress in the inclusion of deaf culture.

*“I am worried because my mother will grow old in the future, and I’m thinking about how difficult it will be when I want to access a health care service, and she will no longer be able to accompany me” (E3, P43).*

The experiences and emotions lived by some deaf individuals have led them to respond to adversity by asserting their independence and autonomy, demanding that health services and hearing individuals provide the necessary accommodations as established by law.

*“During the coronavirus period, when people spoke, they were wearing masks, and they asked me, ‘Where is your mother? Isn’t she accompanying you?’ I said, ‘No! I came alone.’ They didn’t know what to do […] I showed them what the law said, and they didn’t know how to respond. It was a very difficult situation” (E5, P20).*

Regarding the legislation governing the health care context in Chile and its current application in the daily lives of deaf individuals, the interviewees pointed out the lack of concern among health services about providing interpreters, also noting the difficulty in enforcing Law 20.422 on the inclusion of persons with disabilities.

*“We must be strong and assertive about this, and go deeper into the law, because there are no penalties when the law is not enforced—no fines, none. This needs to be strengthened; a decree is needed that establishes fines and ensures compliance, so that people are held accountable for doing their job” (E5, P32).*

There is a perceived inequality in the treatment between deaf and hearing individuals, with some even identifying the migrant population as receiving higher priority.

*“A Chinese person, a Haitian person, when they arrive at a hospital, the nurses rush to assist and help them; they try to communicate and make an effort and everything. But if a deaf person arrives with their child, everyone pretends not to notice—no one wants to attend to them” (E5, P37).*

The interviews also revealed the stance adopted by deaf individuals in their struggle to enforce the law, which involves filing administrative complaints and even considering legal action. However, it was noted that not all deaf individuals are able to exercise this right.

*“I believe there need to be more complaints. We have been coming for a long time, and this has been delayed... Yes, we need to be respected, for the staff to show respect. But it has to be collective, and I believe being united in that is much better” (E3, P27).*

*“Deaf people, for example, some do not know how to read or write; their language is sign language. So how are they going to write a complaint letter to the OIRS? That has to be in writing. That’s where the system fails—it’s an abuse against us. It’s difficult and very sad as well; there are many adjustments missing” (E5, P32).*

Finally, a comparison was made regarding the health care experiences of deaf individuals abroad, highlighting the respect for deaf people’s rights in other countries, in contrast to the limited inclusion within the Chilean health care system.

**Proposals for Improving the Care of Deaf Individuals**

The informants shared ideas for changes to improve their health care, emphasizing the presence of interpreters in health centers as the highest priority adjustment, since this enables effective and timely communication. They also recognized the importance of incorporating LSCh into the professional training of health care students, as well as training health center staff—regardless of their role—so that the information provided is accurate and focused on health-related issues.

*“There should always be an interpreter at the hospital as soon as a deaf person arrives, but the responsibility lies with the hospital, not with the deaf person” (E1, P34).*

*“That doctors and the staff there also know or use sign language. They are professionals, so they can ask focused questions relevant to what they are doing” (E1, P34).*

*“The first thing is to teach them empathy, to do activities with people, to explain what accessibility barriers mean, to put themselves in the other person’s place—that is very important” (E5, P30).*

Services such as the Office of Information, Complaints, and Suggestions (OIRS, by its initials in Spanish), which are available to all users to submit complaints and suggestions, are managed in written form. Interviewees suggested adapting these services to sign language to ensure accessibility for deaf individuals.

*“For example, in the OIRS, change what is written into a video in sign language, so it can be sent to the hospital, and the staff can translate it into Spanish. Because there are deaf people who do not know how to write in Spanish, and when they feel this way, they remain in that situation for a long time, and that affects their mental health” (E5, P32).*

## Discussion

Deaf individuals in Chile face greater social vulnerability, reflected in lower economic income, educational attainment, and employment opportunities. Moreover, they have greater health care needs compared to the general population, as evidenced by the higher number of visits to general practitioners, mental health specialists, and other medical specialists^10^. While deaf individuals do make use of the health care system, it is essential to further explore and understand their access experiences, particularly within the dimensions of non-discrimination, physical accessibility, economic accessibility, and access to information, all of which present specific limitations and challenges.

Among the reported challenges to adequate health care access are communication problems and barriers associated with feelings of mistrust and fear, as well as environments that do not foster satisfactory communication. In addition, deaf individuals demonstrate limited health knowledge due to difficulties in accessing information, highlighting the urgent need for health literacy^11^.

In this regard, Chilean health services do not provide the use of LSCh, forcing deaf individuals to rely on written language and seek support from third parties, thereby highlighting the fact that they cannot receive care without the presence of a companion. In cases where they attend alone, they are asked to communicate in writing, and in some cases, care is even denied—violating Law No. 20,584, which regulates the rights and duties of individuals in relation to actions associated with their health care^12^.

As a result, a situation of unequal treatment and marginalization arises, particularly affecting women in the areas of sexual, reproductive, and gynecological health^11^. Deaf individuals emphasize the need for sign language interpreters to be provided by the State, while also highlighting the importance of health care personnel being proficient in LSCh. From their perspective, this would be the only way to achieve full inclusion for deaf individuals. Along these lines, training and raising awareness among health care staff about deaf culture are recognized as essential measures to improve awareness of barriers and to provide optimal care^13^.

Consequently, structural changes are required—including the development of facilities, training programs, education, and online services—as well as funding to invest in and implement strategies that contribute to improving equity in health care^13^,^14^. Thus, the limitations in the dimensions of physical and economic accessibility indirectly influence the other dimensions of access, even if not explicitly.

Deaf individuals face considerable social pressure due to the lack of understanding and the multiple obstacles they must overcome in their daily lives. It is therefore essential to consider the difficulties they encounter in accessing health care, which are often marked by limitations and barriers that contribute to stigmatization, as well as self-stigmatization, deeply affecting their mental well-being^15^.

It is recommended that initiatives to improve access for deaf individuals be grounded in prior research that identifies their specific needs. The support of those involved—both deaf individuals and health care professionals—is fundamental. In addition, sufficient time is needed to become familiar with the implemented strategies, establish connections, and, above all, build trust, as deaf individuals may at times experience distrust toward the system^16^.

To ensure nursing care that is appropriate for each patient, it is necessary to understand the uniqueness of each individual and adapt care according to their specific needs. Deaf culture is one among many in today’s society and must be respected by the nursing team—from understanding its language to recognizing the cultural differences that translate into a distinct worldview. Adjustments must be implemented respectfully and professionally by trained personnel, such as a sign language interpreter, who should be available in the health care facility and integrated into the care team, as they play a crucial role in the life of the sign language user by connecting them with hearing individuals. Consequently, it is the duty of health personnel to learn to work with such support and to be trained in Chilean Sign Language in order to become less dependent on the interpreter’s role and thereby provide culturally competent care.

There is an urgent need for a model of care that provides guidelines and standards to achieve fully accessible and effective communication with deaf individuals, while also improving the cultural and linguistic competence of health care professionals^17^.

The lack of studies describing the experience and satisfaction of patients with disabilities makes it difficult to compare results with other regions of the country, as such information is not available, thereby revealing a significant knowledge gap regarding deaf individuals.

Research such as this is necessary to shed light on the real public health problems faced by people with disabilities, uncovering the perspective of this population. The main limitation of this study was the small number of participants and its focus on a specific territorial context, which limits theoretical transferability.

## Conclusions

Deaf individuals represent a population whose health care experiences remain largely unknown. Nevertheless, their complexity and heterogeneity are recognized in every respect, including economic and educational backgrounds, their use of their own language, their culture, and their need to be fully integrated into society. These aspects and this diversity keep them united and organized in their pursuit of full inclusion within society, despite the difficulties they face in accessing health care. Challenges are highlighted in relation to physical spaces, communication strategies, care, literacy, training, and cultural and human competence that foster humane and compassionate treatment.

## References

[1] Organización Mundial de la Salud (2024 ). Sordera y pérdida de la audición.

[2] Departamento de evaluación y estudios servicio nacional de la discapacidad (Senadis) (2023 ). III estudio nacional de la discapacidad.

[3] Biblioteca del Congreso Nacional de Chile ( 2023). Ley 20422. Establece normas sobre igualdad de oportunidades e inclusión social de personas con discapacidad.

[4] Organización de las Naciones Unidas ( 2000). El derecho al disfrute del más alto nivel posible de salud..

[5] Ministerio de Salud (Minsal) ( 2022). Plan nacional de salud auditiva y cuidado del oído para Chile 2021-2023.

[6]  Campos   V,  Cartes-Velásquez   R (2022 ). Facilitador Intercultural Sordo en salud para Chile: Análisis de la agenda política a propósito de la Política de Salud de Migrantes Internacionales. Rev Bras Políticas Públicas.

[7]  Ciuffardi  J ,  Sepulveda   T, Bisso  C ,  Daners   P,  Barrios   C ( 2021). Experiencia de las Personas Sordas en la Atención de Salud. Rev Confluencia.

[8]  Schütz   A ( 1972). Fenomenología del mundo social. Introducción a la sociología comprensiva.

[9]  Villa-Velásquez   J ( 2015). “Base de datos de entrevistas asociadas a la atención y acceso a salud de personas sordas”. Mendeley Data, V1.

[10]  Fuentes-López   E,  Fuente  A  ( 2020). Access to healthcare for deaf people: a model from a middle-income country in Latin America. Rev Saude Publica.

[11]  Kuenburg   A,  Fellinger   P,  Fellinger   J ( 2016). Health Care Access Among Deaf People. The Journal of Deaf Studies and Deaf Education.

[12] Ministerio de Salud ( 2012). Ley 20.584: Regula los derechos y deberes que tienen las personas en relación con acciones vinculadas a su atención en salud. Subsecretaría de Salud Pública.

[13]  Morisod  K ,  Malebranche   M,  Marti   J,  Spycher   J,  Grazioli   VS,  Bodenmann   P ( 2022). Interventions aimed at improving healthcare and health education equity for adult d/Deaf patients: a systematic review. Eur J Public Health.

[14] Ryan  C ,  Kushalnagar   P ( 2018). Towards Health Equity: Deaf Adults' Engagement in Social e-Health Activities and e-Communication with Health Care Providers. J Health Commun.

[15]  De Clerck   GAM,  Willems   R (2023 ). Structural stigma in mental health care for deaf and hard of hearing people: the perspectives of care users and caregivers. Tijdschr Psychiatr.

[16]  Smeijers   A,  van den Bogaerde   B,  Ens-Dokkum   M,  Oudesluys-Murphy   AM ( 2020). Specialized outpatient clinic for deaf and hard-of-hearing patients in the Netherlands: Lessons learned in an attempt to improve health care. J Eval Clin Pract.

[17]  Hill   C,  Deville  C ,  Alcorn   S,  Kiess   A,  Viswanathan  A ,  Page   B ( 2020). Assessing and Providing Culturally Competent Care in Radiation Oncology for Deaf Cancer Patients. Adv Radiat Oncol.

